# Impact of Cooperative Learning and Project-Based Learning through Emotional Intelligence: A Comparison of Methodologies for Implementing SDGs

**DOI:** 10.3390/ijerph192416977

**Published:** 2022-12-17

**Authors:** Alba Lozano, Roberto López, Fernando J. Pereira, Carolina Blanco Fontao

**Affiliations:** 1 Department of Mining, Topography and Structures, Area of Mining Prospecting and Research, University of Leon, 24007 León, Spain; 2 Department of Mineralogy, Petrology and Applied Geology, Faculty of Earth Sciences, University of Barcelona (UB), Martí Franquès s/n, 08028 Barcelona, Spain; 3 Department of Applied Chemistry and Physics, Area of Physical Chemistry, University of Leon, 24007 León, Spain; 4 Department of Applied Chemistry and Physics, Area of Analytical Chemistry, University of Leon, 24007 León, Spain; 5 Department of General and Specific Didactics and Theory of Education, Area of Didactics of Experimental Sciences, University of Leon, 24007 León, Spain

**Keywords:** project-based-learning, cooperative learning, sustainability, affordable energy, SDGs, science teaching-learning, education for sustainable development, climate change, emotions

## Abstract

Education for sustainable development (ESD) is a holistic and transformative form of education that seeks action-oriented pedagogy using self-directed learning, participation, and collaboration, among other aspects, and is suitable for developing active methodologies. Since affective-emotional aspects can contribute in the teaching-learning process, this work studies, through a case study, the comparison of the influence of two active methodologies: Cooperative Learning (CL) and Project-Based Learning (PBL) in student emotions and learning processes, as well as their awareness of ESD. For that purpose, a survey was conducted at the fourth secondary level in the science laboratory, subjected to the innovation project e-WORLD, which developed the content of the 7 and 13 Sustainable Development Goals (SDGs) from the 2030 Agenda. Results of ANOVA and Tukey’s tests carried out showed that both methodologies improved skills and knowledge related to climate change and energy, and triggered major positive emotions in students. Furthermore, CL allowed students to acquire more individual and group responsibility than communication skills developed with PBL. It is necessary to continue working on the involvement of students in these methodologies in order to improve their social skills and to reveal life changes towards more socio-sustainable ones.

## 1. Introduction

During the last few decades there has been a technological evolution, which has forced us to adapt to new technologies. Consequently, content studied at high school level has been readjusted to the development of society and its needs. Thus, today, we can understand education as a constantly changing process (formal, non-formal, and informal). It is aimed, through knowledge, attitudes, and values, at promoting a global citizenship that generates a culture of solidarity committed to the fight against poverty and exclusion, as well as to the promotion of human and sustainable development [1,2].

In this sense, education for sustainable development (ESD) can be understood as a holistic and transformative education form, addressing content, the environment, and learning outcomes. Therefore, it not only integrates content in relation to climate change, poverty, and sustainable consumption into the curriculum, but also creates interactive, contextualized, and learner-centered teaching and learning contexts. It seeks a transformative and action-oriented pedagogy, and is characterized by aspects such as self-directed learning, participation, and collaboration, the development of reflective capacity, inter- and transdisciplinary problem orientation, and the creation of links between formal and informal learning. Only such pedagogical approaches can boost key competencies needed to foster sustainable development [3].

This pedagogical approach has been gained relevance and prominence, and has been transferred to different areas and institutions in the belief that the education system cannot and should not be oblivious to the challenges posed by the climate emergency taking place on our planet. Schools and high schools must become a place of stewardship and care for our environment, to become the engines of a culture based on environmental sustainability, social cooperation, and developing programs for sustainable lifestyles, as well as promoting recycling and interactions with green spaces and the more-than-human world.

Consequently, the new Spanish National Organic Law 3/2020, of December 29 (LOMLOE) [4], proposed to collect content for sustainable development and citizenship established by the 2030 Agenda. This Agenda includes, among other topics, education on ecological transition and local action to address the climate emergency and energy crisis, established in several of the Goals of Sustainable Development (SDGs). The new law, materialized in the curricula of the different autonomous communities, requires adapting to the new demands posed by social, economic, and environmental evolution, while it is essential to promote the achievement of education for sustainable development through the SDGs of the 2030 Agenda [5].

Transforming educational plans has become a great challenge for politicians and teachers, with the aim of improving society as a whole and finding synergies between intellectual development and the re-humanization of education for personal and social services. Scientific literacy, in this aspect, has become a key international objective in order to face the current challenges of humanity [6]. For that reason, today, educational projects including these concepts have considerably increased. These projects are based on achieving greater empowerment for the student, giving meaning to their education through involvement in the problems of society, while the student must be associated with the real world context; in this case, climate change [7,8].

In this way, the student acquires meaningful learning when he/she is involved in society’s problems and is made to participate in them. The changes introduced in the teaching-learning models allow students to develop the skills and competences that are part of their reality through the design, implementation, and evaluation of projects connected to their life and interests.

An example of these new projects is the e-WORLD innovation project, owned by the Repsol Zinkers Foundation and intended for secondary school students. This project works specifically on SDG 7, affordable and non-polluting energy, and SDG 13, climate action, with the aim of raising awareness of the need to globally reduce CO_2_ emissions. This program is carried out by applying two active methodologies: project-based learning (PBL) and cooperative learning (CL) [9]. It approaches the teaching-learning process as contextualized through a real problem in which the students have to become involved in the decision-making process to face the problem, which causes the project to intrinsically embrace SGD4: quality education.

From the *constructivist theories* framework, Ausubel defines meaningful learning as active learning, produced by the relationship between previous knowledge, already acquired by the student, and new knowledge [10]. This learning is enhanced through the development of active methodologies, such as project-based learning (PBL) and cooperative learning (CL). Both methodologies cause the student to become actively participative in the learning process, while the teacher acts as a guide in the process [11,12]. The PBL promotes student skills for problem solving: autonomous learning, critical attitude, communication, cooperation, and decision-making [13]. It also develops creativity, autonomy, and motivation [14]. In addition, at the beginning of the learning process, clear and challenging criteria and guidelines are established to increase their involvement, where the teacher is a facilitator of learning [15].

CL is a methodology based on the organization of a classroom, where the students work in groups due to the skills and resources exchange among classmates [16]. In addition, teamwork learning promotes interpersonal relationships and respect [17]. Recent studies have shown that the use of active methodologies in the classroom not only favors meaningful learning, but also allows the development of personal, social, and professional skills, or even critical spirit [18,19,20], which are necessary to develop the skills that are evaluated in the PISA report [21]. Therefore, a study of the use of active methodologies and their evolution according to the changes in education takes on special importance to ensure meaningful learning and learning by competencies.

In recent years there has been growing interest in emotional intelligence and the teaching-learning process in relation to the skills developed by the students [22]. Emotional intelligence has its origins in the Theory of Multiple Intelligences as described by Garner [23], in which he defines eight types of independent intelligences. Two of them, interpersonal and intrapersonal intelligences, are defined as the ability to attend to other people and the ability to have a coherent image of oneself, respectively. In the mid-1990s, the term emotional intelligence was established, defined by Goleman as “the ability to recognize our own feelings and those of others, to motivate ourselves and manage emotionality in ourselves and in interpersonal relationships” [24].

Results regarding science teaching have shown that emotional intelligence improves learning outcomes [25,26]. Mellado et al. [27] focused on the study of the emotions of science teachers and students with the aim of designing intervention programs for the control and self-regulation of their learning through knowledge of their emotions. In a similar trend, another study on the evolution of the emotions of physics and chemistry students throughout the three Compulsory Secondary Education (ESO) courses showed that positive emotions in relation to science decreased after each course [28]. Along the same lines, the results obtained in [29] showed that students expressed mainly negative emotions in relation to science content.

In addition to the effects on cognitive aspects, emotions are important because they act as predictors of behavior. Weiner’s attribution theory makes it possible to explain why students experience positive or negative emotions towards subjects based on their successes or failures [30]. Those students that present academic failures in science subjects have felt negative emotions that cause rejection towards scientific learning. On the other hand, those who have achieved academic success have experienced positive emotions that generate attraction and motivation towards this same type of learning [31]. In this way, the use of active methodologies could provoke positive emotions that cause student interest in science to last, while enhancing meaningful learning.

Emotional intelligence also allows learning by competences. Its development can improve social skills and interpersonal interaction in students, which could be included in emotional competence [32]. This competence favors problem solving and prevents violent behavior among teenage students through emotions management [33]. In addition, neurodidactics, understood as a discipline that studies the mechanisms that the brain uses to optimize the educational process, has made it possible to explain, for example, why active learning methodologies based on projects (PBL) and cooperative learning favor student learning by promoting their interests and motivations [34], and by acquiring skills and competencies [18].

For the reasons previously stated, it has been considered necessary to study emotions in projects based on the implementation of the SDGs in the curricular content of secondary school students. In this way, it could be known if the learning situations through active and innovative methodologies, associated to a real problem in the student environment, favors motivation and interest in experimental sciences, as well as increases meaningful learning.

## 2. Objectives

This work aims to study the influence of two active methodologies (CL and PBL) on emotions and on the teaching-learning process, as well as the awareness of students in ESD working on the 7 and 13 SDGs using a case study. In order to achieve this objective, the specific defined objectives are:

- Objective 1: Analyze the different impact among skills developed caused by the two active methodologies used in the research (CL and PBL).
- Objective 2: Study the impact that methodologies have on the emotions of science students.
- Objective 3: Estimate the relationship between emotions and active methodologies.
- Objective 4: Evaluate student results in the learning environment and energy contents through 7 (affordable and clean energy) and 13 (climate action) SDGs.

## 3. Materials and Methods

### 3.1. Implementation of Active Methodologies through the e-WORLD Innovation Project

The e-WORLD project develops two of the Sustainable Development Goals (SDGs) of the 2030 Agenda: SDG 7, affordable and clean energy, and SDG 13, climate action. Concomitantly, SDG 4, education quality, has also developed thanks to this project as active methodologies, and thus, promoting different learning opportunities and experiences [5]. It is addressed to high school students and it is based on two active methodologies: cooperative learning (CL) and project-based learning (PBL). The program consists of five chapters, with a common thread being to help the virtual protagonist, Alex, to reduce global CO_2_ emissions. Thus, students are divided into groups and must solve several problems in order to achieve the global objective of the project. The teacher acts as a guide in challenges on air quality, sustainable mobility, climate change, and energy mixing, providing support when necessary. All the information regarding the project teaching guide is published on the Repsol Zinkers website [9]. The e-WORLD project was carried out over twelve sessions of a subject of regional configuration of Castilla y León of Science Laboratory of 4th ESO [35]. This subject is based on student skills development that encourages them to become capable of exploring facts and phenomena; in this case, related to energy, analyzing problems, and organizing relevant information.

### 3.2. Research Instrument and Participants

The research methodology was based on an exploratory case study, where twelve teaching sessions of two science classes were performed using the CL and PBL active methodologies. The study used a non-probabilistic convenience sample, made up of 38 students in the fourth year of ESO from two different classrooms (4 °C and 4 °D). The students ages ranged between 15 and 17 years, with an average of 16 years. There was only one repeating student; 42% of the sample were men and 58% were women. There was no control group since it was considered a specific sample for the case study of the e-WORLD project. The students belonged to the public center Ordoño II High School (León, Spain). The research was carried out during the 2020/2021 academic year.

A descriptive-comparative and non-experimental quantitative design was selected for this study. For this purpose, a questionnaire was chosen as a research tool with the aim of exploring the impact that the used active methodologies presented in the students’ learning process and their emotions. Two questionnaires were distributed and answered by all the students in May 2021. The study of methodology and emotions were cross-sectional (conducted once the project had finished), while the study of learning results was longitudinal (pre-post test).

The first questionnaire was based on the original questionnaire reported by Dávila-Acedo in [29] and adapted to the e-WORLD experience (Appendix A), following the requirements of our sample and on suggestions from the Repsol Zinkers Foundation [9]. The questionnaire was divided into two main blocks and an extra initial block that collected demographic characteristics of the students (class of the student, age, sex, and if they repeated). The questionnaire was mixed and consisted of a total of 29 questions. For the first draft of the questionnaire items, a group of experts in the teaching and learning of experimental sciences (two university professors belonging to the area of didactics of experimental sciences and the area of chemistry) met with the purpose of adapting the questions of the reference questionnaire to the e-WORLD project. The questions were chosen and a second draft was designed. Subsequently, a pilot test was carried out with a twofold objective: to determine the need to modify, add, or eliminate questions, and to detect possible mistakes or limitations. The questionnaire was revised and reformulated for distribution to the students. 

Questions in block 1 used a 5-point Likert-type scale with values to measure the degree of perception/opinion/agreement about the statements made, where 1 was equivalent to “not at all/barely” and 5 was “a lot/totally agree”. Questions in block 2 used the multiple-choice option.

The first block *Benefits of active methodologies* evaluated the contribution of PBL and CL methodologies in terms of student learning. It was divided into eight categories (A–H). A to F were based on the main elements that the two active methodologies (CL and PBL) are specific [15,17]. Categories A, B, and C described cooperative learning skills, and categories D, E, and F described the project-based learning capabilities (see Table A1 in Appendix A):

A. Positive interdependence: referred to the process of acquiring a dual responsibility for personal and group learning.
B. Personal and individual responsibility: referred to the student’s own awareness of their individual learning and the enhancement of their cognitive abilities.
C. Self-assessment: referred to the reflection of the students towards their actions in the activities carried out and their results.
D. Face-to-face interaction: referred to the fact that learning provides a pleasant climate where classmates must help each other and become involved with the group, without discriminating against classmates.
E. Interpersonal and group skills: referred to communication and respect among students, approaching new colleagues, or conflict resolution capacity.
F. Feeling of leadership and involvement in the work: one of the main characteristics of project-based learning is that students take the lead in their learning.

The two extra categories from block 1 (G and H) referred to student learning and awareness regarding energy and climate related to SDGs 7 and 13.

G. Refers to the process of being aware of the necessity and commitment to contribute to minimizing climate impact.
H. It is more technical and refers to learning concepts about energy and climate change, referring to SGD 7 and 13.

The second block *Emotions caused during the project* was also based on the questionnaire reported by Dávila-Acedo in [29]. It evaluated the emotions experienced by the students during the learning process carried out through active methodologies. This block consisted of 12 multiple choice questions. The categories in this block were equal to block 1, and were indicated by the corresponding letter (see Table A2 of Appendix A).

The second questionnaire was a test owned by Repsol Thinkers Foundation, who developed the e-WORLD project, and its information is protected [9]. It consisted of a 24-question test on climate change, energy, and its transformation. Each question had three possible answers and only one was correct. The scores obtained were obtained by only counting the correct answers. Students answered it twice: before and after the project, and it was carried out to assess their evolution in the learning process to study possible differences between pre and post test in terms of the knowledge acquired (see results in Appendix A). 

The internal consistency coefficients (Cronbach’s Alpha) of both questionnaires were 0.868 (active methodologies) and 0.810 (learning experience), indicating good reliability of the instrument, although the study sample was relatively low.

### 3.3. Data Processing and Statistical Analysis

The main questionnaire (questionnaire 1) was developed using Google forms survey administration software. Responses of Likert blocks were numerically converted as follows: “Not at all”: 1, “Hardly”: 2, “Sometimes”: 3, “Frequently”; 4; “Totally”: 5. 

All raw data were converted to absolute and relative frequencies, respectively. The software used for calculations was R-studio software (2022 release). 

An analysis of variance (ANOVA) was carried out to study the differences between the used methodologies [36]. When these differences were significant, a Tukey’s SHD test was performed to conduct binary comparisons between the groups and, therefore, to know which of the variables presented the highest influence [37,38].

In addition, for the sake of evaluating the relationship between emotions and categories, a Principal Component Analysis (PCA) was carried out [39,40].

In order to study the differences between the knowledge acquired pre and post test, an ANOVA was performed.

Finally, the significance levels taken in the present study were: *p* > 0.05, not significant, and *p* ≤ 0.05, significant difference.

## 4. Results

Table 1 shows the results obtained from the student answers in relation to the skills developed from cooperative learning and project-based learning methodologies. The absolute frequency distribution indicated a majority of responses rated between medium and higher scores corresponding with agree and totally agree in the Likert scale.

### 4.1. Analysis of Differences between Methodologies and Categories

The results of the ANOVA performed to compare the means of the values from the different categories and the methodologies used in this study are described below. In this sense, the results are presented in Table A3. Different tests (nine in total) were carried out to compare the different variables of the study. When the ANOVA showed significant differences, Tukey’s HSD test was performed (six in total).

The ANOVA test regarding methodologies as the variable object of study indicated the existence of statistically significant differences between the two active methodologies (*p* = 0.009, Test 1). Tukey’s HSD test indicated that the methodology CL is more attractive for students than PBL (*p* = 0.009; Table A4, Test 1). 

The ANOVA test including categories as the variable studied also indicated statistically significant differences among categories of each methodology (*p* = 0.008) and between the CL and PBL methodologies (*p* = 0.001) (Table A3, Test 2). Then, a Tukey’s HSD test was performed to compare all of the categories from both methodologies. The results showed significative differences for four categories (*p* < 0.05, Table A4, Test 2). The differences were summarized as:

- F: Leadership feeling—B: Individual responsibility: the students better develop the individual responsibility with CL methodology than leadership feeling with PBL.
- B: Individual responsibility—A: Positive interdependence: students feel more development in positive interdependence than individual responsibility when work with CL methodology.
- F: Leadership feeling—E: Interpersonal/group sills: students develop more interpersonal than leaderships skills when considering BPL methodology.

### 4.2. Relation between Active Methodologies and Student Emotions

Table 2 shows the students’ emotions according to cooperative learning and project-based learning methodologies among each category. Generally, the absolute frequency distribution indicated that students felt more positive than negative emotions in all categories selected for the two methodologies and for SGDs learning aspects.

When statistical analysis was carried out, the ANOVA test indicated statistically significant differences between methodologies (*p* = 0.02) and emotions (*p* ≤ 2 × 10^−16^) (Table A3, Test 3) so the active methodologies presented an impact in emotions expressed by students.

The ANOVA test was repeated in order to assess if methodologies impacted more positive or negative emotions. The ANOVA test used methodologies and emotions as variables, where emotions were encoded with 1 or 0 for positive or negative (expressed as feeling variable in the test), respectively. Results showed that there were statistically significant differences between methodologies (*p* = 0.047) and between positive or negative emotions (*p* ≤ 2 × 10^−16^) (Table A3, Test 4). Post-hoc analyses using Tukey’s HSD test for significance between methodologies and positive or negative emotions indicated that CL (*p* = 0.047) impacted more positively for students than PBL, as they expressed more positive emotions (*p* = 0.000) than negative with respect to this methodology (Table A4, Test 3).

Considering the categories and emotions, the ANOVA test indicated statistically significant differences between both of them (emotions *p* ≤ 2 × 10^−16^ and categories *p* = 0.000; Table A3, test 5), concomitant to Test 1. Again, the characteristic skills developed thanks to each methodology were expressed by different emotions from students.

**Figure 1:**
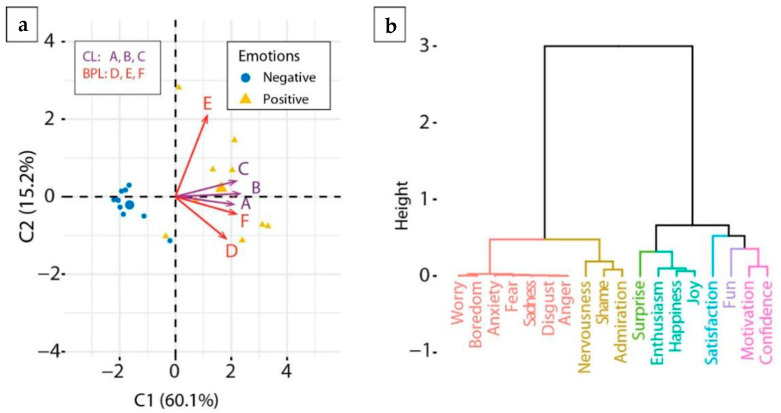
(**a**) PCA result of categories as variables and emotions as individuals. Both CL variables (A, B, C) and BPL (D, E, F) are associated with positive emotions distribution. (**b**) Emotion clustering.

Principal Component Analysis (PCA) between emotions and categories were performed in order to group categories as a function of emotions. According to PCA results (Figure 1a), the first dimension represented 60% variance of emotions and the majority of categories (with the exception of admiration) were linked to positive emotions. The cluster dendrogram of emotions resulted in two first divisions, which mainly corresponded to positive and negative emotions, respectively, and was concomitant to what was observed in the PCA results (Figure 1b).

Finally, a Tukey’s HSD test was performed in order to differentiate the categories according to the main emotions. The categories showed significant differences for the learning process, with *p* < 0.05 (Table A4, Test 4). Only four categories showed significant differences, from which all differences were obtained between F category “leadership feeling” and other three categories:

- F (leadership feeling)—A (positive interdependence): students feel that gain interdependence when working CL but lose individual responsibility with BPL.
- F (leadership feeling)—E (interpersonal skills): students feel lose leadership but develop interpersonal skills in BPL.
- F (leadership feeling)—D (face to face interaction): students feel lose leadership but develop more face to face in BPL.

### 4.3. Active Methodologies Impact in Teaching-Learning Process

The results reported in Table 1 show the students answers according to SGD learning (categories G and H). The absolute frequency distribution indicated a majority of responses ranged between 4 and 5 scores. 

The ANOVA test did not show statistically significant differences (*p* = 0.329) (Table A3, Test 6) between SDGs learning. In contrast, by considering the questions as variables yielded a statistically significant difference among the questions of both SGDs learning (*p* = 0.020) (Table A3, Test 7). However, the Tukey’s HSD test results showed that questions 2 and 4 were statistically more favorable than 1 and 3, which meant no statistical differences between the questions of the G and H categories (*p* = 0.733, Table A4, Test 5). These results suggested a similar learning of energy and climate change concepts by students.

The impact that energy and climate change learning through SGD had on student emotions was evaluated by the ANOVA test. The results indicated the existence of statistically significant differences between emotions (*p* < 2 × 10^−16^), thus playing an important role, but no significant differences were found for categories G and H (*p* = 0.694) (Table A3, Test 8). 

The ANOVA test was performed to also elucidate which kind of (positive or negative) emotions were statistically predominant. The results showed that positive and negative emotions were significant different (*p* < 2 × 10^−16^) (Table A3, Test 9) and the Tukey’s SHD test indicated that positive emotions were predominant, regardless of the category (*p* = 0, Table A4, Test 6). The Tukey’s HSD test for comparing pairs of emotions indicated that satisfaction (*p* = 2.42 × 10^−14^) and surprise (*p* = 3.17 × 10^−14^) were the emotions most positively expressed by the students when learning about SGDs 7 and 13, respectively.

#### Learning Results: Pre and Post Test

**Figure 2:**
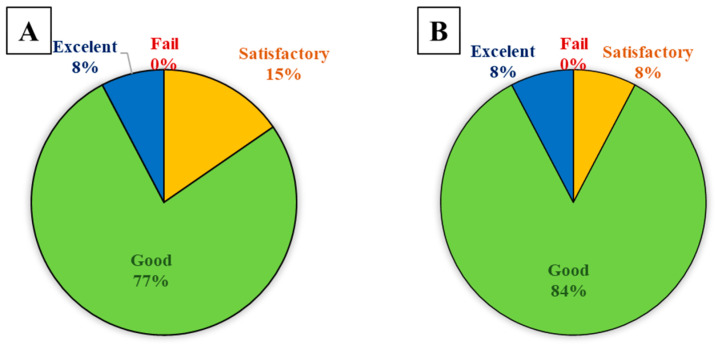
Percentage of students who obtained the indicated grade range. Fail (grade < 5), Satisfactory (5 ≤ grade < 7), Good (7 ≤ grade < 9), Excellent (grade ≥ 9). (**A**). Pre test, (**B**) Post test.

The ANOVA test was performed to assess differences between mean scores in pre and post tests of energy and climate content performed by students, (A) before and (B) after the innovative project. Results (Appendix A, Table A5) suggested statistically significant differences between the scores of pre and post test (*p* = 0.015), being a mean increase of 0.577 points with an improvement range of 0.11 and 1.04 points (Figure 2). Thus, a learning improvement was found thanks to the active methodologies. 

## 5. Discussion

Objective 1 of the study aimed to analyze the different impact of the skills developed by working with the two active methodologies while learning; a very positive impact was observed in both of them. These results corroborate the improvement in science education through active methodologies already described by many authors in the last few decades [41,42]. When comparing the two methodologies carried out in this study, the differences observed were greater. In fact, working with the CL methodology allowed the highest development of personal/interpersonal and interdependence skills in students. Further, CL allowed students to mature in terms of their commitment to individual and group responsibility for the learning process. Furthermore, it gave students the skills to understand and respect each other, which allowed them to be comfortable with their classmates. This is especially important today, as classrooms are increasingly more culturally diverse [43,44]. In contrast, BPL skills were less developed than in CL, which could be interpreted as a necessity to enforce critical and communicative attitudes and decision-making capacity, as there are aspects that still have to be fully developed in students [45]. 

In relation to Objective 2, which aimed to evaluate the impact of the methodologies of emotions in science students, a predominance of positive emotions was observed over negative emotions. The challenge faced by teachers regarding reducing negative student emotions of students in physics and chemistry subjects is being overcome [27,29,46] and, as a consequence, students’ attitudes towards science have already improved [47].

The majority of positive emotions indicated that the learning process was favored. This was especially found when using CL. For instance, González-Gómez et al. [48] explained how the intervention of the CL methodology had improved the management of students’ fear, as well as moral emotions, promoting an improvement in learning. This methodology developed a strong motivational process of belonging to a group and the ability to self-manage help among colleagues [17], which could explain the positive emotions such as happiness and satisfaction. A majority of students felt self-confident, capable of regulating their emotions and generating a positive experience of the project [49]. These results were similar to those described by Aguilar et al. [50], as they observed that chemistry students in the fourth year of science developed greater motivation in the classroom when designing a specific program for that subject using PBL and CL. 

Although negative feelings were minor in terms of presence, it is important to discuss what kind of reasons can explain their appearance. Emotions of fear, boredom, or disgust were considered to deactivate the teaching-learning process [51]. The lack of criticism and communication, more related to BPL skills, could reflected negative emotions expressed by students. These emotions are considered unpleasant but exciting, according to Dávila-Acedo et al. [27], and they promote confusion, but, when they generate tension, they can be considered as activating emotions of the teaching-learning process [51].

Related to the Objective 3 of the study, where we tried to estimate the relationship between emotions and active methodologies, the results showed that both methodologies influenced student emotions. In addition, students expressed a majority of positive emotions, such as happiness, satisfaction, or surprise, contrary to those generalized by students in science studies with traditional methodologies [29]. Emotions mark the development of student skills within the learning methodology, with cooperative learning and positive emotions being the most predominant. Although the main expressed negative emotions, nervousness and embarrassment, may generate tension, together they can act as activating emotions in the teaching-learning process. These results indicate that it is necessary to continue working on the development of social skills in students, since they are fundamental for their professional future. In current work environments, a change in teammates is frequent, which must be faced with emotional regulation due to the association between work productivity and being emotionally competent [32]. 

Focusing on Objective 4, related to learning about the environment and energy, the results showed the same trend that other previous researches had found in the literature, in which CL was applied to all school stages [52] or when PBL was used to teach robotics in secondary school and improve STEAM (science, technology, engineering, arts, and mathematics) learning [53]. Active methodologies can favor the capacity for meaningful learning through an increase in student motivation, thanks to encouraging positive emotions [54] and the ability to learn [18,19].

### Limitations and Future Research Lines

The limitation of this study was the sample size. However, it can be justified by the kind of research performed, based on an exploratory case study methodology. In the literature, we found many case studies with similar small sample sizes [55,56,57]. For instance, Brownell et al. [58] concluded that even small sample sizes are important, as the results could provide an indicator of potential directions for improving teaching and learning. Another work justifies case studies from sample sizes of six individuals as, in education, parameters expansion cannot be controlled from the beginning of the research [59].

As future research, it is proposed to further the study of the interconnection between emotions and learning, in addition to seeing how other current methodologies, such as STEM projects and service learning, influence emotions and favor ESD. It is important to know whether the involvement of students in these methodologies produces changes in the life habits of adolescents towards more socio-sustainable habits. Following this idea, it is also important to avoid territorial risk of bias in the study. Thus, for future research and the distribution of the same or new questionnaires to other high schools, we should first analyze what kind of data may produce territorial bias risk, similarly to the study by Bellantuono et al. that presented a comparison between the university academic rankings affected by bias [60]. A possible way to overcome bias could be by creating a more extensive questionnaire with information regarding contextualization of social and economic conditions to students [61].

## 6. Conclusions

From the need to promote the paradigm shift of formal education towards a model that promotes the achievement of the SDGs and the goals described in the 2030 Agenda, innovative projects applicable to the classroom are essential to carry out this achievement and to facilitate the process from teachers to students [1,3,62]. Thus, in recent years, there has been an increase in the number of projects focused on this purpose, making it more necessary to prove their effectiveness through evidence from educational research [63,64].

After the results obtained in the present work, it can be concluded that CL and PBL methodologies are efficient for the improvement of skills and knowledge related to climate change and energy in the students, provoking positive emotions in a higher frequency, thus favoring meaningful learning. The use of active methodologies minimized the negative impact on learning in science subjects related to sustainable development. Therefore, CL and PBL active methodologies can be potential resources for the emotional and cognitive improvement of the science teaching-learning process in secondary schools.

The comparison of the two methodologies revealed that CL allowed students to acquire personal and group responsibility in learning to a higher extent than PBL, which allowed them to develop their own learning objectives, expressing positive emotions that favor learning. As for the PBL, students had lower achievement in terms of social skills, such as critical attitude, interpersonal communication, and decision-making, although it did not directly impact either negative emotions or the learning objectives from the ESD approach perspective.

## Figures and Tables

**Table 1:** Absolute frequencies of responses on active methodologies (block 1).

	Methodologies/Learning	Cooperative Learning								Project Based Learning					SGD-Learning			
	Categories	A	A	A	B	B	B	C	C	D	D	E	E	F	G	G	H	H
	Question	1	2	3	4	5	6	7	8	9	10	11	12	13	14	15	16	17
Likert scale	1	0	0	0	0	0	0	0	0	0	0	1	0	1	2	1	0	0
	2	3	3	6	0	1	0	0	1	3	0	1	1	6	2	0	3	1
	3	2	6	8	2	5	4	11	3	2	2	4	6	11	7	4	3	3
	4	18	8	12	14	10	10	13	22	10	19	14	22	11	16	14	22	13
	5	15	21	12	22	22	23	14	12	23	17	18	9	9	11	19	10	21
	Sample sum	38	38	38	38	38	37	38	38	38	38	38	38	38	38	38	38	38

Source: own elaboration.

**Table 2:** Absolute frequencies of responses on active methodologies.

	Cooperative Learning				Project Based Learning				SGDs Learning			
Categories	A	A	B	B	C	C	D	D	E	F	G	H
Questions	18	19	20	21	22	23	24	25	26	27	28	29
Joy (+)	13	5	10	8	3	3	10	9	9	7	7	9
Self-confidence (+)	12	7	9	11	6	10	9	2	5	16	8	3
Happiness (+)	5	5	16	9	2	6	8	12	6	7	4	12
Admiration (+)	0	2	4	7	4	4	3	1	0	6	1	1
Satisfaction (+)	6	12	8	20	9	5	19	17	1	7	24	15
Enthusiasm (+)	7	4	2	6	4	8	7	6	8	8	3	11
Surprise (+)	0	7	9	0	2	2	5	5	13	0	8	9
Motivation (+)	13	11	7	9	6	13	4	8	3	8	3	11
Fun/Enjoyment (+)	20	9	1	0	3	7	2	2	5	4	0	3
Boredom (−)	0	4	0	0	0	1	0	1	3	1	1	0
Anxiety (−)	2	2	0	0	1	0	0	1	0	1	0	0
Fear (−)	0	0	0	1	2	1	0	0	1	0	0	0
Disgust (−)	0	0	0	0	0	0	0	0	1	0	0	0
Sadness (−)	0	0	1	1	0	0	0	0	2	1	1	0
Anger (−)	2	0	1	0	0	0	0	0	1	0	1	0
Nervousness (−)	4	5	0	2	14	8	0	0	3	1	0	0
Concern (−)	2	2	0	1	0	0	0	2	2	0	0	0
Embarrassment (−)	2	2	1	4	9	1	0	0	2	0	0	0
Total responses	88	77	69	79	65	69	67	66	65	67	61	74
Sample sum	38	38	38	38	38	38	38	38	38	38	38	38

Source: own elaboration.

## Data Availability

Data is contained within the article. Additional data are available on request from corresponding author.

## Appendix A

**Table A1:** Self-made questionnaire for students based on Dávila et al. (2017), (Google forms application) [29].

STUDENTS’ DATA:							
Age: Sex: man/woman Are you a repeater student?							
yes/no							
BLOCK 1. BENEFITS OF ACTIVE METHODOLOGIES							
Asses from 1 to 5 how much you agree with the following statements in relation to the work done during the e-WOLRD project. (1. Not at all/hardly agree—5. Totally agree/very much.							
**Nº**	**CAT.**	**ITEMS**	**1**	**2**	**3**	**4**	**5**
1	A	Among all of us we have been able to manage the tasks to finish them on time. Doing homework is a group goal, so my success is everyone’s.					
2	A	There was no group leader in the activities, we have worked together to complete the tasks.					
3	A	We were all interested in learning and so have helped each other.					
4	B	I have assumed my responsibility in what I had to do in each task.					
5	B	I’ve asked my teammates for help when I’ve got stuck on what to do on homework.					
6	B	I have helped a colleague when I thought I could help him or when they have asked me for help in the group.					
7	C	During the activities, everyone has contributed with their ideas and knowledge, so I have improved my communication skills.					
8	C	During the performance of the tasks, I have been able to listen to my colleagues and accept criticism.					
9	D	Working in a group and confronting different points of view has made us obtain better results.					
10	D	My role group has worked well and coordinated, we have managed to learn new content on energy and sustainability.					
11	E	Working in a group has allowed me to get to know my colleagues better.					
12	E	We have been able to agree to carry out the task when there have been different ideas.					
13	F	I have felt like a protagonist, since learning depended on my interest and abilities, which has also favored my creativity.					
14	G	Involvement in the project has allowed me to be aware of the challenges of the energy transition and climate change, and I would like to create concern in my family and friends.					
15	G	What I have learned has helped me to become more aware of my habits, and to contribute to have a more sustainable planet.					
16	H	I have learned new energy contents that are useful in my day to day life.					
17	H	The methodology to learn new content has been easier than in a normal class.					

**Table A2:** Emotions caused during the project based on Dávila et al. (2017) [29], (Block 2). Indicate the emotions that you think best fit EACH SITUATION of those listed below.

			Positive Emotions									Negative Emotions								
			Cheerful	Self-Conficdent	Happy	Amazed	Satisfied	Excited	Surprised	Motivated	Fun	Boring	Anxiety	Fear	Disgusting	Sad	Angry	Nervous	Worried	Embarrased
18	A	Working together as a team and has made me feel…																		
19	A	When I had to solve the calculation of energy mix (simulator) with my team I have felt…																		
20	B	When I have had some difficulty in challenges on climate change/air quality, energy mix calculations, etc., but a member of the group has helped me, I have felt…																		
21	B	When I have been able to help a colleague to do a task I have felt…																		
22	C	When I have made oral presentations I have felt…																		
23	C	When I have participated in debates in climate change or in air quality I have felt…																		
24	D	Learning new things from my own work and research have made me feel…																		
25	D	The project results obtained by working as a team have made me feel…																		
26	E	Changing work teammates has made me feel…																		
27	F	Having a role in the project has made me feel…																		
28	G	When I can understand scientific news and those related to climate change and the energy transition, I feel…																		
29	H	When I have carried out practical work, which I can also apply to daily life, with my team I have felt…																		

**Table A3:** ANOVA results.

	Test nº	Categories	F Value	Pr (>F)
Active methodologies and skills	Test 1	Methodology	6.964	0.0086
	Test 2	Methodology	7.166	0.0077
		Category	4.617	0.0011
Active methodologies and emotions	Test 3	Methodology	5.386	0.0206
		Emotions	35.172	<2 × 10^−16^
	Test 4	Methodology	3.961	0.0469
		Feeling	227.68	<2 × 10^−16^
	Test 5	Emotions	36.392	<2 × 10^−16^
		Category	4.641	0.00035
Active methodologies and learning process and emotions	Test 6	Category SGDs Learning	0.957	0.329
	Test 7	Questions	3.367	0.02
	Test 8	Category	0.0155	0.694
		Emotions		<2 × 10^−16^
	Test 9	Category	0.117	0.733
		Feeling	740.334	<2 × 10^−16^

**Table A4:** Tukey’s SHD results.

		Comparison	Diff	P Adjusted
Active methodologies and skills	Test 1	CL-BPL	0.2461	0.0086
	Test 2	F-B	−0.6012	0.0175
		B-A	0.4	0.0299
		F-E	−0.5789	0.0432
Active methodologies and emotions	Test 3	CL-BPL	0.0317	0.0469
		(+)—(−)	0.2954	0
	Test 4	F-A	−0.0919	0.0027
		F-E	−0.0993	0.0122
		F-D	−0.0841	0.0257
	Test 5	H-G	−0.009	0.7331
Active methodologies and learning process and emotions	Test 6	(+)—(−)	0.894	0

**Table A5:** E-word test Results.

	Results			Results	
Student Number	Pre-Test	Post-Test	Student Number	Pre-Test	Post-Test
1	5	7.5	21	9.2	8.8
2	5.8	7.5	22	8.3	6.3
3	7.5	8.8	23	7.5	7.5
4	6.7	8.8	24	8.3	7.1
5	8.3	6.7	25	7.5	8.3
6	8.3	7.9	26	8.3	6.7
7	7.5	8.8	27	5.8	7.9
8	7.5	7.9	28	7.5	5.8
9	5	8.3	29	9.2	7.9
10	5	7.9	30	6.7	7.9
11	6.7	7.5	31	6.7	7.5
12	6.7	8.8	32	7.5	9.2
13	9.2	7.1	33	6.7	6.3
14	7.5	8.3	34	8.3	6.7
15	7.5	7.5	35	8.3	8.8
16	6.7	8.3	36	8.3	9.6
17	7.5	7.9	37	8.3	8.8
18	7.5	9.2	38	6.7	7.5
19	6.7	6.7	39	7.5	8.8
20	5	7.9			
